# Data Integration Methods for Phenotype Harmonization in Multi-Cohort Genome-Wide Association Studies With Behavioral Outcomes

**DOI:** 10.3389/fgene.2019.01227

**Published:** 2019-12-10

**Authors:** Justin M. Luningham, Daniel B. McArtor, Anne M. Hendriks, Catharina E. M. van Beijsterveldt, Paul Lichtenstein, Sebastian Lundström, Henrik Larsson, Meike Bartels, Dorret I. Boomsma, Gitta H. Lubke

**Affiliations:** ^1 ^Department of Psychology, University of Notre Dame, Notre Dame, IN, United States; ^2^Netherlands Twin Register, Department of Biological Psychology, Vrije Universiteit Amsterdam, Amsterdam, Netherlands; ^3^Faculty of Behavioural and Movement Sciences, Amsterdam Public Health Research Institute, Vrije Universiteit Amsterdam, Amsterdam, Netherlands; ^4^Department of Medical Epidemiology and Biostatistics, Karolinska Institutet, Stockholm, Sweden; ^5^Gillberg Neuropsychiatry Centre, University of Gothenburg, Gothenburg, Sweden; ^6^School of Medical Sciences, Örebro University, Örebro, Sweden; ^7^Amsterdam Neuroscience, VU University Amsterdam, Amsterdam, Netherlands

**Keywords:** phenotype harmonization, genome-wide association studies, latent variable modeling, data integration, consortia

## Abstract

Parallel meta-analysis is a popular approach for increasing the power to detect genetic effects in genome-wide association studies across multiple cohorts. Consortia studying the genetics of behavioral phenotypes are oftentimes faced with systematic differences in phenotype measurement across cohorts, introducing heterogeneity into the meta-analysis and reducing statistical power. This study investigated integrative data analysis (IDA) as an approach for jointly modeling the phenotype across multiple datasets. We put forth a bi-factor integration model (BFIM) that provides a single common phenotype score and accounts for sources of study-specific variability in the phenotype. In order to capitalize on this modeling strategy, a phenotype reference panel was utilized as a supplemental sample with complete data on all behavioral measures. A simulation study showed that a mega-analysis of genetic variant effects in a BFIM were more powerful than meta-analysis of genetic effects on a cohort-specific sum score of items. Saving the factor scores from the BFIM and using those as the outcome in meta-analysis was also more powerful than the sum score in most simulation conditions, but a small degree of bias was introduced by this approach. The reference panel was necessary to realize these power gains. An empirical demonstration used the BFIM to harmonize aggression scores in 9-year old children across the Netherlands Twin Register and the Child and Adolescent Twin Study in Sweden, providing a template for application of the BFIM to a range of different phenotypes. A supplemental data collection in the Netherlands Twin Register served as a reference panel for phenotype modeling across both cohorts. Our results indicate that model-based harmonization for the study of complex traits is a useful step within genetic consortia.

## Introduction

Multi-study consortia and large-scale meta-analyses are the status quo for genome-wide analyses of complex traits (Evangelou and Ioannidis, 2013; Pedersen et al., 2013; Reitveld et al., 2014). Combining data from different studies presents an additional challenge when behavioral, psychological, or other complex phenotypes have been measured by different means across the studies. The most practical and widely used phenotype scoring approach is forming a sum or mean score of the available measures in each cohort (Wray et al., 2007; Bath et al., 2010; Pappa et al., 2016). However, sum scores overlook the systematic measurement differences that are brought about by different questionnaires. Sum scores of different item sets may not capture the same aspects of the behavior, so their use introduces phenotypic heterogeneity and reduces power in genome-wide association studies (GWAS; van den Berg et al., 2014). The current paper utilizes an integrative data analysis (IDA) framework for phenotype harmonization that can provide benefits for consortium-based GWAS meta-analyses by improving precision in phenotype measurement (Curran and Hussong, 2009). To quantify these benefits, we conduct a simulation study to assess the power to detect the effect of a genetic variant on a behavioral outcome that is modeled by IDA-based phenotype harmonization. In addition, we illustrate the IDA approach to harmonizing behavioral phenotypes.

IDA is a broad framework that holds great potential for improving the phenotype measure in GWAS meta-analyses because it is essentially *model-based* phenotype harmonization. The IDA framework allows researchers to adjust for measurement differences across studies, which is usually not possible when conducting meta-analyses of summary statistics. The common practice of forming sum scores of questionnaire scales is based on the often implicit assumption that the individual items available in each cohort measure the same phenotype across studies, which rarely holds in studies of complex behavioral outcomes. Different sets of items usually evaluate different aspects of a behavioral phenotype; and there are often measurement differences across countries or cultures, age groups, or different raters (Hudziak et al., 2003; Bartels et al., 2007; Jak, 2017). Typical approaches to phenotype harmonization, such as collapsing or rescaling response categories, are not sufficient when there are substantial differences underlying phenotype measurement (Gatz et al., 2015).

Phenotype precision has been demonstrated to improve statistical power and precision for genetic association tests. For example, removing poor measurement items can reduce heterogeneity in the phenotype, thereby increasing the signal associated with genetic variants (Laurin et al., 2015). In a different study, Xu et al. (2015) showed that fitting a complex psychometric model to mental health data led to larger single-nucleotide polymorphism (SNP) effects than performing a GWAS on a sum of mental health items. The study suggested that psychometric models more accurately reflect complex traits than the sum score, which ignores possible multidimensional subtypes of a trait. Indeed, simulations have shown that accounting for multidimensionality of a behavioral outcome with latent trait models can increase power in a GWAS compared to a sum score (van der Sluis et al., 2010). An additional advantage of psychometrically harmonizing behavioral phenotypes lies in the fact that, for many behaviors, subtypes of a given trait can have different levels of heritability (Ligthart et al., 2005; Yeh et al., 2010). This indicates that different SNPs may be acting on the different trait subtypes. Therefore, a single phenotype score ignoring dimensionality muddies strong genetic signals with weak or non-existent ones, resulting in less overall power than would be present if a subtype were accurately scored.

In IDA, item- or subscale-level data from different consortium partners are concatenated into one dataset. Psychometric modeling (Cattell, 1952; Lawley and Maxwell, 1963) allows items from different cohorts to contribute differentially to the scoring of the underlying trait which represents the phenotype and has the same metric across cohorts. The advantage of IDA is the flexibility to adjust for measurement differences in the measurement model specification such as rater, sex, and/or cohort differences. An inherent challenge of IDA is that combining the item-level data from different cohorts usually introduces a large amount of missing data due to the fact that not all cohorts use the same questionnaires. To illustrate, suppose cohort A uses questionnaire X, whereas cohort B uses questionnaire Y. Responses of questionnaire Y would be missing in cohort A and the reverse would be true for cohort B. IDA measurement models require the presence of overlapping items to adequately link data across all participants (Hussong et al., 2013). Collecting a supplemental sample with complete data on all items can help alleviate this problem (Hussong et al., 2013; Gatz et al., 2015). In this paper, the supplemental sample is called a phenotypic *reference panel.*

IDA has been used previously to combine different versions of cognitive batteries, personality measures, and alcohol use data across multiple studies (van den Berg et al., 2014; Xu et al., 2015; Marcoulides and Grimm, 2017). Many IDA approaches used a Rasch item response theory (IRT) model, a latent trait model that requires simplifying assumptions and specifies equivalent measurement across study (McArdle et al., 2009; Gatz et al., 2015; Marcoulides and Grimm, 2017). Other IDA models directly evaluate the differences of measurement properties of the pooled items. Curran and Hussong (2009) and Curran et al. (2014) proposed a moderated non-linear factor analysis (MNLFA) model that allows all of the item measurement parameters to vary across a set of covariates, such as study membership and country of origin. In a similar approach, Bauer et al. (2013) integrated data across multiple raters (e.g., mother and father ratings). This model extracts a single phenotype score while filtering out rater influences. The model is based on an adaptation of the bi-factor model, a classic psychometric model in clinical research (Holzinger and Swineford, 1937). The IDA model proposed in this paper is built upon the bi-factor model where a general factor represents the sought after phenotype that is common to multiple studies. Differences across cohorts are modeled in a set of specific factors. In addition to separating the common phenotype from cohort-specific influences, the bi-factor integration model also eliminates measurement error from the phenotype factor score.

This paper is structured as follows. A brief review of factor analysis and structural equation modeling (SEM) is presented. Practical issues for meta-analysis in GWAS consortia and the potential advantages of a phenotype reference panel are discussed. A model specifically suited for IDA in multi-study GWAS, termed the bi-factor integration model (BFIM), is then presented. A series of simulations demonstrate how IDA can increase power to detect a genetic effect when phenotype reference panel data are available. A demonstration of the bi-factor integration model for two large datasets of aggressive behaviors in 9-year-old children is also presented. The implications of these results for behavior genetic consortia are discussed, as well as limitations of this approach and future directions.

## Methods

### Factor Analysis Models

Behavioral and mental health phenotypes such as intelligence or depression are not directly observable but instead are measured with multiple questionnaire items. The observed items are individual indicators of an underlying construct. The observed indicators are designed to capture the different aspects of the construct, and item responses are considered as manifestations of the true trait. Latent variable models capture the information that is common across multivariate outcomes (i.e., shared variance), considered the underlying latent trait or factor (Bollen, 1989). For example, aggression is a trait that is not measured directly, but researchers administer multiple questions that pertain to different aspects of aggressive behaviors or attitudes. Individuals with higher levels of true aggression are expected to score higher on the items. Additionally, the underlying factor fully accounts for the covariance of the items; once the aggression factor is accounted for, the items are conditionally independent from each other.

The factor analysis model is a direct implementation of this line of thought. Let *y* *_ij_* represent a response to item *i* (*i *from 1, 2, …, *p*) for person *j* (*j*from1,2,…,*N*) Assuming a single latent variable underlies a set of observed, continuous item responses, the model can be written as

(1) $$y_{ij}=\nu_{i}+\;\lambda_{i}\eta_{j}+\varepsilon_{ij}$$

where *v* *_i_* represents an item intercept, *λ* *_i_* is an item loading (or slope) parameter, *η* *_j_* represents the latent factor score for person *j*, and *ɛ* *_ij_* is an error term. The latent factor is assumed to be normally distributed as *N* (*α,ψ*) and the error is normally distributed as *ɛ* *_ij_*∼*N*(0,*σ* ^2^). Individuals are assumed to be independent from each other, and the items are conditionally independent given *η* *_j_*. To identify the model, one item intercept and loading must be fixed to zero and one, respectively, or the factor variance must be fixed at one (Lawley and Maxwell, 1963; Bollen, 1989).

### The Bi-Factor Integration Model

Several models developed for IDA can be used to test measurement differences in the item parameters one at a time for multiple items over multiple covariates (Curran et al., 2014). In the GWAS integration scenario, however, the only interest is in reducing the noise in the phenotype score introduced by differences in measurement across cohorts. If one can reasonably assume that the available questionnaire items are all indicators of the same phenotype, then the target trait of interest is simply a single common factor underlying the full item set. If the items used by the different cohorts tap into similar aspects of the phenotype and have similar measurement properties, then the sum score model is expected to work reasonably well. However, a simple unidimensional factor analysis model may not fit well if items used in the different cohorts measure more or less severe aspects of the phenotype, if cohorts differ with respect to raters, or if items have different meanings across cultures.

In this paper we propose a bi-factor model integration model (BFIM) that increases precision in the estimated target trait by modeling additional information specific to different questionnaires or cohorts. The BFIM is a special case of the factor model in equations 1 and 2 with multiple factors, which can be written as

(2) $$y_{ij}=\nu_{i}+\;\lambda_{ig}\eta_{jg}+\overset{K}{\underset{k=1}{\sum }}\lambda_{ik}\eta_{jk}+\;\varepsilon_{ij}$$

where *η* *_jg_* represents a *general* factor for person *j*, labeled T for the target trait, and there is an associated factor loading for all items on *T, λ* *_ij_* *. η* *_jk_* represents a *specific* factor *k* (*k=*1,2,…,*K*) that only subsets of items load onto, with *λ* *_ik_* corresponding to item loadings on the *k* *^th^* specific factor.

Similar to the general factor analysis model, the underlying factors are assumed to be normally distributed as *η* *_jk_* *∼N* (*α* *_k_*,*ψ* *_k_*), and the error is normally distributed as *ɛ* *_ij_*∼*N*(0,*σ* *^2^*). The bi-factor model specification also requires constraints to identify the model. The bi-factor model is identified by specifying that the factors follow a standard normal distribution (Gibbons and Hedeker, 1992). Further, each item loads onto the general factor and only one additional specific factor; otherwise, the model is not identifiable (Gibbons and Hedeker, 1992). Consider a case with eight total items and four items loading onto each of two specific factors. The matrix of factor loadings and vector of latent factors are then (3)

(3) $$\Lambda =\left[\begin{array}{ccc} \lambda_{1g} & \lambda_{11} & 0\\ \lambda_{2g} & \lambda_{21} & 0\\ \lambda_{3g} & \lambda_{31} & 0\\ \lambda_{4g} & \lambda_{41} & 0\\ \lambda_{5g} & 0 & \lambda_{52}\\ \lambda_{6g} & 0 & \lambda_{62}\\ \lambda_{7g} & 0 & \lambda_{72}\\ \lambda_{8g} & 0 & \lambda_{82} \end{array}\right];\eta =\left[\begin{array}{c} \eta_{jg}\\ \eta_{j1}\\ \eta_{j2} \end{array}\right]$$

**Figure 1:**
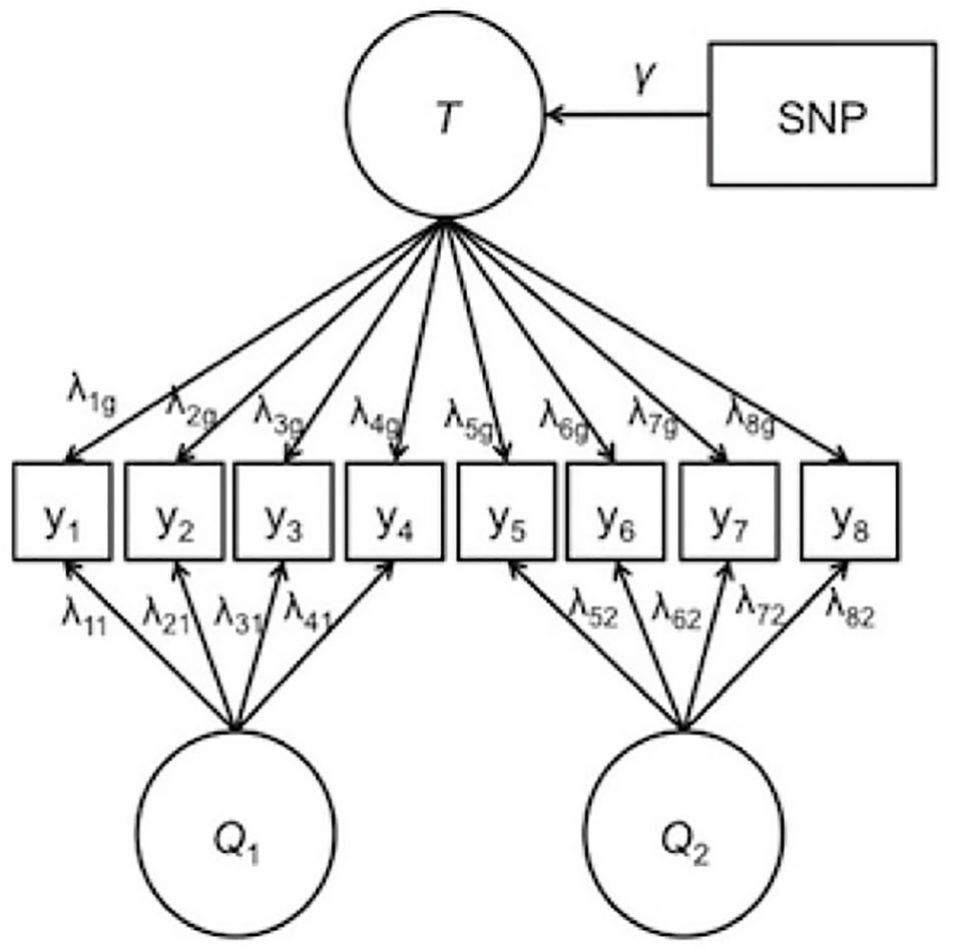
Example of the bi-factor integration model. *T* represents the general trait. Q_1_ and Q_2_ are the specific factors corresponding to questionnaire 1 and questionnaire 2, respectively. *λ* represents the factor loading, *γ* represents the effect of the single-nucleotide polymorphism on the general trait. Thresholds and error terms are not depicted.

Orthogonality is imposed on the factors such that the specific factors are completely independent of the general factor and of the other specific factors. Due to this independence specification, the general factor captures the target trait of interest, and the specific factors pull out the residual covariance among item *subsets* that is not captured in the common factor. For IDA across multiple studies, modeling the specific factors can follow known differences across the studies. For example, specific factors may be modeled from items that originate from the same questionnaire, the same study, or the same country of origin. By explicitly modeling these sources of heterogeneity, the precision of the general factor is increased. Improved phenotypic measurement, in turn, is likely to increase the precision in the SNP association coefficients. **Figure 1** depicts a bi-factor integration model for two different questionnaires with a general factor, two specific factors, and a SNP effect. In **Figure 1**, the specific factors are labeled *Q* *_1_* and *Q* *_2_* corresponding to questionnaire 1 and questionnaire 2, respectively.

The target factor scores can be computed from the bi-factor integration model and then used by partnering studies in a consortium to conduct a parallel meta-analysis. Using computed factor scores as outcomes in association tests should result in more precision in each study, therefore increasing precision in the average effect size. Reducing the standard error of the estimated

$$\bar{\beta }$$

increases the power of the hypothesis test that the effect is significantly different from zero. Factor scores are not estimated in the latent variable model, but computed *post hoc* using fixed model parameters. There are multiple approaches for computing factor scores, such as regression scores, Bartlett scores, likelihood-based expected a-posteriori scores, and Bayesian plausible values (Muthén and Muthén, 1998-2017). Certain types of factor scores used as dependent variables can lead to bias in the regression coefficients (Grice, 2001; Skrondal and Laake, 2001). Devlieger et al. (2016) demonstrated that different methods of factor score calculations have similar rates of statistical power, so regression factor scores were calculated for this study.

Combining data across studies with disjoint measurement variables creates a dataset with systematic missing data. Modeling an underlying factor in combined data is therefore also a missing data problem. The three most commonly assumed missing data mechanisms are missing completely at random (MCAR), missing at random (MAR), and missing not at random (MNAR; Enders, 2010; Little and Rubin, 2002). When data are MCAR or MAR, full information maximum likelihood estimation is known to provide correct inferences (Rubin, 1976). The MAR mechanism states that missing values of *Y* are independent of the observed values of *Y* after taking other variables in the analysis into account. In the IDA case, the missing data are accounted for by study or cohort membership, and missingness is independent of scores on the trait of interest. Maximum likelihood estimation is well developed for SEM and confirmatory factor analysis approaches (Allison, 2003; Asparouhov and Muthén, 2010; Enders, 2010).

### Genome-Wide Association Studies Meta-Analysis

Common practice for GWAS in a consortium is for each consortium partner to conduct association tests on a sum of all available items. The resulting regression coefficients are then meta-analyzed. In this case, the phenotype score is the sum of the items, which can also be written in the context of the factor model:

(4) $$SS_{j}=\overset{p}{\underset{i=1}{\sum }}y_{ij}=\;\overset{p}{\underset{i=1}{\sum }}(\nu_{i}+\;\lambda_{i}\eta_{j}+\varepsilon_{ij})$$

The general association test (prediction model) for the sum score is simply

(5) $$SS_{j}=\beta_{0}+\overset{Q}{\underset{q=1}{\sum }}\beta_{q}x_{qj}+\epsilon_{j}$$

where *x* *_qj_* is the genotype available for person *j* at locus *q,* and β_q_ is the regression coefficient for SNP *q.* The primary interest is in the *p*-value and statistical significance of *β* coefficients corresponding to SNPs. In a meta-analysis, these coefficients are combined across the different individual analyses and their significance is re-evaluated. Consortia commonly use fixed-effects meta-analysis with inverse-variance weighting or the *Z*-score method (Evangelou and Ioannidis, 2013). Fixed effects meta-analysis requires the assumption that there is one true population value for the effect of interest. Given this, if the sum scores *SS* *_j_* are calculated from different items across study, additional error will be introduced into the regression weight of a SNP on the heterogeneous sum scores.

Sum scores are straightforward, easy to compute, and easy to interpret. They do, however, come with drawbacks. With sum scores, the item uniquenesses and measurement errors are summed up along with the portions of the item relating to the true trait. This will increase the variance of the outcome relative to the underlying structural variance of the trait, leading to inflated standard errors for ${\hat{\beta }}_{q}$ in each study. Additionally, estimating the factor loadings in a measurement model permits each item to contribute to the latent factor with different weights, reflecting the fact that different questionnaire items are often not equally good indicators of an underlying trait. A sum score implicitly assigns equal weight to all items. Regressing a harmonized trait score on a set of SNPs should result in a meta-analysis of regression coefficients that are more directly comparable.

### Genome-Wide Association Studies Mega-Analysis

In general, it is preferable to fit the integrated measurement model and simultaneously conduct genetic association tests. The ideal scenario is to carry out a mega-analysis with SEM in which the measurement model for *η* and the regression of *η* on *x* is estimated simultaneously (Bollen, 1989). While this may be computationally difficult for a full genome-wide search with millions of SNPs, it is certainly possible for cases in which a few hundred or even thousand candidate SNPs are identified (e.g., Xu et al., 2015). Furthermore, recent methodological advances have increased the computational feasibility of SEMs in GWAS, such as with genome-wide structural equation modeling (GW-SEM) (Verhulst et al., 2017). In the context of the bi-factor integration model, the covariate effect is truly expressed on the target trait factor *T* *_j_* rather than the indicators themselves. This is specified as the structural portion of the SEM model. For observed covariates *x* *_qj_* (q from 1, 2, …,*Q*), the prediction model is written

(6) $$T_{j}=\overset{Q}{\underset{q=1}{\sum }}\gamma_{q}x_{qj}+\zeta_{j}$$

where *T* *_j_* the target (general) trait score for person *j,* γ*_q_* is the regression coefficient for the *q* *^th^* covariate, and ζ_j_ is a residual disturbance term. In a GWAS, the γ*_q_* of primary interest is the one associated with a SNP, but controlling covariates such as age, gender, and genetic relatedness principal components may also be included.

### Phenotypic Reference Panel

Retrospectively combining independent studies with different instruments often results in a sparse dataset with a high degree of missing data. This can lead to sets of subjects with no common items, resulting in latent variable models that often do not converge using modern estimation approaches for handling missing data. Lack of convergence leads to flawed estimators, if the model is able to provide estimates at all. Typical harmonization treats items that are similarly worded in the different questionnaires as the same item in the combined dataset, thus creating item overlap. This can also destabilize the model, however, if the items are not truly interchangeable.

A better strategy for understanding the relationships among all items is to collect a reference panel with complete data. The reference panel provides information about the association between items not jointly observed within cohorts when different surveys are used. This supplemental sample is critical for providing a link across cohorts and offers a potential gateway for psychometric harmonization through IDA. Similar approaches have been applied for multiple imputation integration, in which measurement models are not used (Carrig et al., 2015; Siddique et al., 2015). When there is available research and theory about a psychological phenotype, exploring a small number of measurement models can offer more precision for subsequent analyses than approaches making no assumptions about structure in the data (Collins et al., 2001). Others have collected a reference panel-type sample and analyzed it separately to evaluate the performance of more conventional phenotype harmonization approaches (Gatz et al., 2015). The ACTION Consortium (Aggression in Children: Unraveling gene-environment interplay to inform Treatment and InterventiON strategies) is actively collecting a *post hoc* phenotype reference panel in order to facilitate the multi-study integration of complex models of childhood aggressive behaviors (Boomsma, 2015).

The collection of the reference panel is imperative for the BFIM (and any measurement model) in the case of insufficient item overlap. In the next section, we present a Monte-Carlo simulation study evaluating the use of the bi-factor integration model compared to sum scores in hypothetical SNP association tests. These simulations provide insight into whether the collection of the reference panel and the extra effort in phenotype modeling are worth the costs.

## Simulation

We conducted a simulation study with the goal of comparing the power of sum score meta-analysis, factor score meta-analysis, and integrated mega-analysis (full data integration model with SNP effect). A multiple imputation procedure was also carried out as an alternative method for handling missing items in the cohorts. The simulation was set up to represent a scenario in which two different studies used two different questionnaires to measure the same trait. Each cohort only had item responses on one questionnaire, with missing data on the items used in the other study. A small reference panel dataset was also included, in which subjects had responses on all items across the two questionnaires.

Data were simulated under four different data-generating models with five different sample size conditions, resulting in 20 total simulation conditions. To evaluate the necessity of the reference panel, data were also generated without a reference panel (with additional subjects added to one or both cohorts). **Table 1** lists the different models and sample size conditions utilized in the simulation study. In the first three sample size conditions, the reference panel makes up ∼4, ∼2.5, and ∼7.5% of the total dataset, with equal sample sizes in the two cohorts. In the fourth condition, unequal sample sizes are introduced into the cohorts. In the fifth condition, the reference panel is increased to make up 10% of the total sample, maintaining similar sizes to condition 1 and 4. For all 20 combinations of model and sample size, 1,000 repetitions were executed in the simulation.

**Table 1:** Various data-generating models and sample sizes used in simulations, resulting in 20 simulation conditions.

	Sample sizes	Data-generating models
Varying simulation conditions	N1: cohort 1 = 5,000, cohort 2 = 5,000, Ref= 400	Model 1: same measurement across item sets
	N2: cohort 1 = 2,500, cohort 2 = 2,500, Ref= 400	Model 2: different levels of item set reliability
	N3: cohort 1 = 7,500, cohort 2 = 7,500, Ref= 400	Model 3: mean and variance differences in cohort-specific factor
	N4: cohort 1 = 2,500, cohort 2 = 7,500, Ref= 400	Model 4: true model is a higher-order model (bi-factor model is misspecified)
	N5: cohort 1 = 4,500, cohort 2 = 4,500, Ref= 1,000	

For all data-generating models, the underlying factors all followed a (marginally) standard normal distribution, and factor loadings were invariant across the reference group and cohort item sets. A SNP covariate accounted for 0.1% of the variance in the general factor, and a second covariate representing biological sex accounted for 20% of the variance in the general factor. Note that this assumes a single population-level effect size of the SNP, the same assumption made in fixed effects meta-analysis (Evangelou and Ioannidis, 2013). It was also assumed that the multiple cohorts originated from comparable populations, meaning that the minor allele frequency of the SNP was the same across groups. In practice, quality control checks with a reliable genotype reference should be conducted for either meta-analysis or mega-analysis approaches, and population stratification should be controlled for.

The genotypes for the SNP were generated with a minor allele frequency of 0.5, such that the data-generating equation for the target trait was

(7) $$T_{j}=0.0447*SNP_{j}+0.8944*X_{2j}+\;\zeta_{j},\;\zeta_{j}\;\sim \;N\left(0,\;0.799\right)$$

leading to a marginal variance of 1 for *T* *_j_*, and the specific factors were orthogonal to *T* *_j_* and each other and were standard normal. Item-level data were then generated from the bi-factor model: (8)

(8) $$y_{ij}=\nu_{i}+\;\lambda_{ig}T_{j}+\lambda_{ik}\eta_{jk}+\;\varepsilon_{ij}$$

where factor loadings *λ* *_ig_* ranged from 0.3 to 0.6, depending on data-generating condition (specific simulation parameters are detailed in Appendix I). The loadings for the general and specific factors were controlled such that the general factor and specific factor collectively accounted for either 60 or 45% of each items’ variance. For example, for factors with marginal unit variance, the explained variance of the factors is $\lambda_{ig}^{2}$+ $\lambda_{ik}^{2}$ . There were eight items total: four items for each cohort, representing a brief item set tapping into a sub-domain of a behavior. Two items across cohorts were also generated with an additional residual correlation, reflecting items across questionnaires that were similar but not *exactly* the same, such as items that might be harmonized based on similar wording in the prompts. The residual correlation for these items was set at 0.6.

### Data-Generating Models

Data were generated for the two cohort-specific item sets under a series of different measurement models These measurement differences were quite mild compared to the potential level of heterogeneity that is often encountered in practice, but this allowed us to examine the effect of increased measurement precision with even a small amount of measurement heterogeneity. All data were generated in R (R Core Team, 2018).

**Figure 2:**
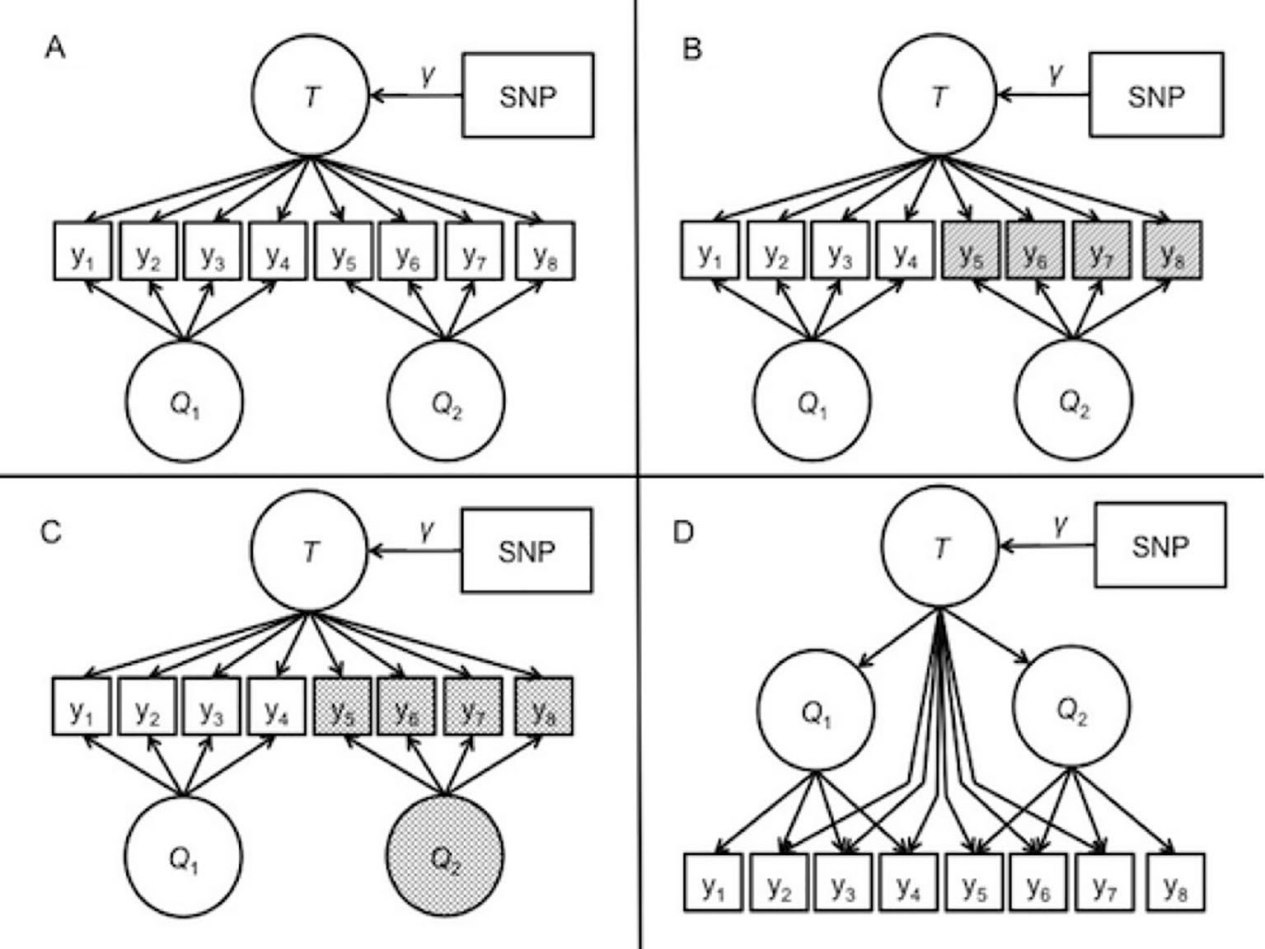
Path diagrams of the data-generating models for the simulation. *T* represents the general trait. Q_1_ and Q_2_ are the specific factors corresponding to questionnaire 1 and questionnaire 2, respectively. **(A)** depicts model 1, representing the ideal measurement case with equal reliabilities for both questionnaires. <br/>**(B)** depicts model 2, in which the items on the second questionnaire have lower reliabilities than the first questionnaire, represented by shading. **(C)** depicts model 3, in which the Q_2_ factor is shaded, representing an increased mean and variance along with reliability differences. **(D)** depicts model 4, a second-order model where the general factor summarizes the covariance among two questionnaire-specific factors, and the general factor additional direct effects on select items.

**Model 1: ideal measurement.** The first model, depicted in **Figure 2A**, represented identical measurement across the two cohorts. This model reflects ideal measurement conditions where the item sets have equal reliabilities. In other words, the factors account for the same amount of variance in the item responses. The factors collectively account for 60% of the variance in the item responses. The general factor accounts for 25.5% of the item response variability, and the specific factors account for 34.5% in the item responses. This contribution breakdown is equal across the two questionnaires.

**Model 2: reliability differences.** The second data-generating model is depicted in **Figure 2**. In the second model, the variance explained by the second set of items was lower than the first set. This reflects an applied scenario in which less reliability in the second measure. To simulate this, the general factor and specific factor accounted for only 45% of the variance in the observed item responses of the second item set, rather than 60% of the variance in the more reliable set. Consequently, one set of four items has residual variance of 40%, but the other item set has residual variance of 55%. This weaker contribution is split between the general and specific factors, resulting in a slightly weaker contribution of the covariate effect to the manifest variables.

**Model 3: mean and variance differences.** The third data-generating model is described in **Figure 2C**. This model featured a larger mean and variance in the specific factor that contributes to the less reliable items of model 2. Rather than having mean of zero and unit variance, the specific factor had a mean of 1 and variance of 3. At the population level, the effect size of the SNP on the general factor remained the same. With increased mean and variance in the specific factor, the proportional contribution of the specific factor on the manifest variables also increased.

**Model 4:** higher-order data-generating model. Model 4 is depicted in **Figure 2D**. The fourth model reflected a scenario where the bi-factor model is somewhat misspecified. The data-generating model was re-formulated as a higher-order factor model in which the covariates influenced a target trait factor, which then influenced two separate cohort factors that finally determined the item responses in each of the cohorts. This model included direct effects from the true factor to some of the item responses. Chen et al. (2006) demonstrated that the higher-order correlated factors model is a constrained version of the bi-factor model. In fact, the correlated factors model with direct effects of the higher-order factor on all items is equivalent to the bi-factor model. In our simulation, some of the items did not include a direct effect from the trait factor to the item, meaning that the BFIM was slightly misspecified to the <br/>generated data.

Each model was used in combination with the five different sample size conditions, and for each model and sample size, 1,000 repetitions were generated to conduct analyses. The codes used to conduct the simulation are attached as a supplementary downloadable folder.

### Analyses

Four different types of analyses were carried out to evaluate the effect of the genetic variant on a trait score across the simulated cohorts. The analyses were designed to compare sum scores in each cohort with the measurement model integration approach utilizing the reference panel. The different analysis procedures are listed below:

1. **Sum score meta-analysis:** The mean score of available items in each cohort and in the reference panel was computed. A meta-analysis of the SNP effect on the mean score in the two cohorts and reference panel was conducted. Meta-analyses were conducted directly in R (R Core Team, 2018).
2. **Factor score meta-analysis:** A BFIM was fitted to the overlapping phenotype information with no genetic variant included. The BFIM models were fitted using Mplus 7.11 (Muthén and Muthén, 1998-2017), and estimation was carried out using maximum likelihood estimation with numeric integration. Numeric integration was needed because of the low rates of coverage for some items. Regression-type factor scores were saved and used as the outcome in association tests within the two cohorts and reference panel and subsequently meta-analyzed. Meta-analyses were computed in R (R Core Team, 2018).
3. **BFIM mega-analysis:** The BFIM was fitted to all of the items to model a harmonized phenotype, and the general factor of the BFIM was regressed on the SNP simultaneously in the structural part of the model. The full SEM mega-analysis models were fitted using Mplus 7.11 (Muthén and Muthén, 1998-2017), and estimation was carried out using maximum likelihood estimation with numeric integration.
4. **Multiple imputation:** Multiple imputation of the individual items is an alternative approach to addressing missingness in the combined data. For a benchmark comparison, we used multivariate imputation by chained equations (MICE, van Buuren and Groothuis-Oudshoorn, 2011) to impute each item from all other items and the covariates. We then summed the items for an imputed sum score as the outcome in mega-analysis association tests. Predictive mean matching, which is the default imputation model of the “mice” package in R^1^, was specified as the imputation model. The default value of 5 iterations was used for each imputed dataset, and 50 datasets were imputed for each to account for the large proportion of missing data. Results from the regression analyses were pooled according to Rubin’s rules to obtain correct standard errors and degrees of freedom (see Rubin, 1987; van Buuren, 2018; for details on pooling, predictive mean matching, and multiple imputation).

A bi-factor SEM was also carried out with complete data (i.e., no missingness) for all participants as a benchmark for the maximum power that could be achieved. To evaluate the utility of the reference panel, the factor score meta-analysis and SEM mega-analysis were also conducted without a reference panel included. In order to fit the models, some overlapping item information must be present. Therefore, the items with large residual correlation across questionnaires were treated as the same item. This is consistent with the practical scenario in which similarly worded items are recoded as equivalent items in the harmonization process.

The primary outcome of interest was the empirical power to detect the SNP effect (i.e., proportion of significant findings). Raw empirical power and empirical power relative to the maximum power under the complete data condition were computed. Type I errors, relative bias, and 95% coverage rates were also recorded. Relative bias is the difference between the true parameter and the average estimate across repetitions divided by the true value.

## Results

The overall results indicated a small-to-moderate advantage to detect the SNP effect for the data integration approach, in general. Under the fourth model data-generating model, in which the bi-factor model is misspecified, the sum score meta-analysis outperforms the factor score meta-analysis, but the BFIM mega-analysis still provides the best overall result.

### Power and Type I Error

**Figure 3:**
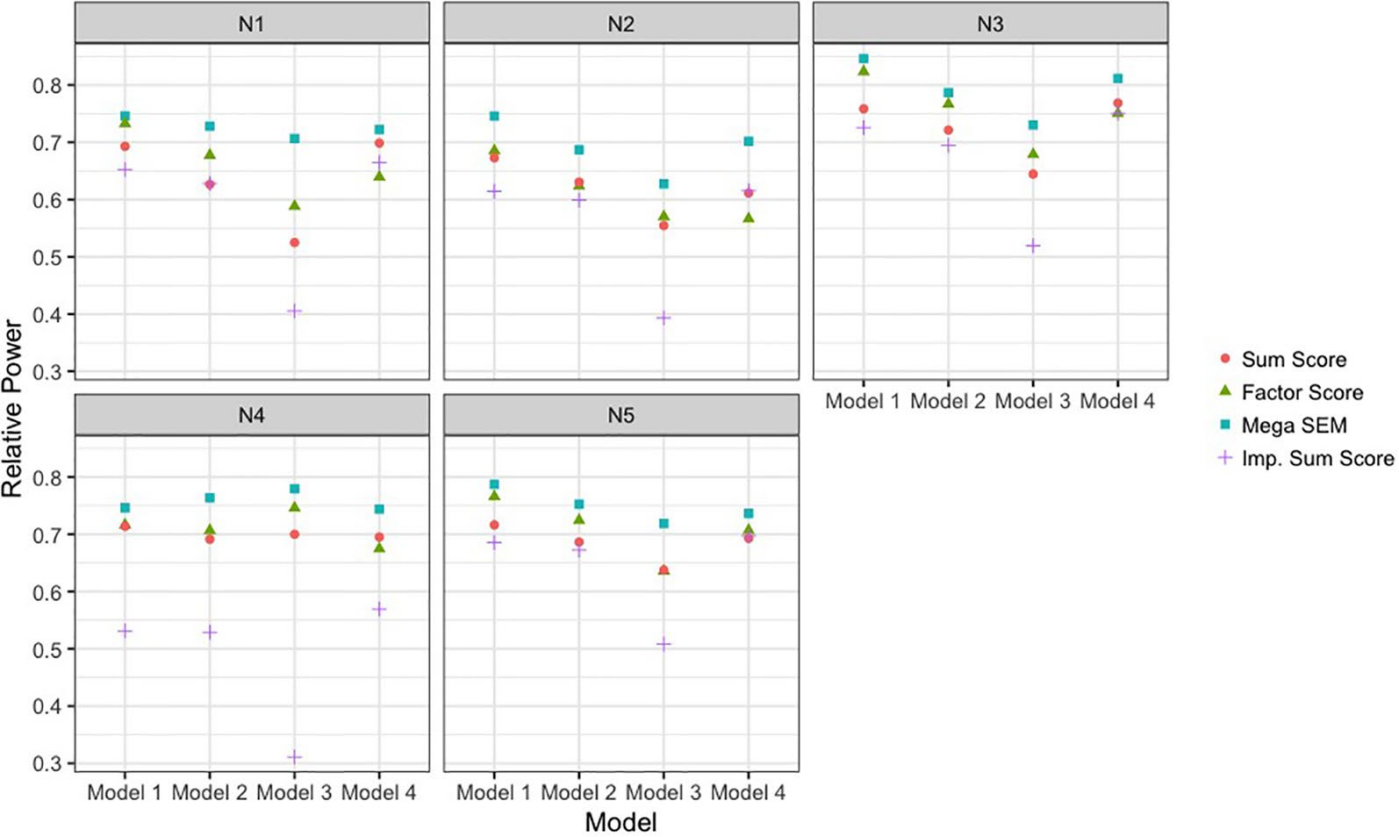
Power to detect single-nucleotide polymorphism effect of each analysis approach relative to complete data power. Results are presented across the different data-generating models and the different sample size conditions detailed in **Table 1**. The analysis approaches were sum score meta-analysis, factor score meta-analysis, mega-analysis with the bi-factor integration mode, and multiple imputation of the missing items. SEM, structural equation modeling; Imp., imputation.

**Figure 3** presents the different rates of power of the sum score meta-analysis, factor score meta-analysis, and BFIM mega-analysis relative to the power obtained with no missing data. Power is plotted as a function of different data-generating model across different panels representing the various sample size conditions. This was included because the raw power is not truly generalizable, as some data-generating models by design had less power to detect the genetic effect even with complete data than others. Therefore, the empirical power relative to the maximal power is more comparable across conditions. **Table 2** presents the raw power rates of each of the methods, as well as type I error rates.

**Table 2:** Empirical power and type I error results with different analysis methods under the four data-generating models and five sample size conditions.

Model	N	Complete data power	Mega SEM	FS meta	SS meta	Impute power	Full T1	Mega T1	FS T1	SS T1	Impute T1
Model1	N1	0.760	0.567	0.557	0.519	0.496	0.046	0.046	0.044	0.040	0.037
Model2	N1	0.710	0.517	0.481	0.445	0.446	0.051	0.044	0.043	0.055	0.039
Model3	N1	0.678	0.479	0.399	0.356	0.275	0.045	0.047	0.071	0.067	0.037
Model4	N1	0.760	0.549	0.486	0.531	0.505	0.049	0.047	0.047	0.059	0.047
Model1	N2	0.480	0.358	0.329	0.323	0.295	0.046	0.040	0.057	0.051	0.033
Model2	N2	0.444	0.305	0.277	0.280	0.266	0.045	0.057	0.064	0.057	0.047
Model3	N2	0.384	0.241	0.219	0.213	0.151	0.048	0.045	0.045	0.044	0.043
Model4	N2	0.466	0.327	0.264	0.285	0.287	0.047	0.051	0.050	0.044	0.051
Model1	N3	0.878	0.743	0.723	0.666	0.637	0.055	0.054	0.042	0.043	0.034
Model2	N3	0.858	0.675	0.658	0.619	0.596	0.045	0.049	0.054	0.048	0.044
Model3	N3	0.816	0.596	0.554	0.526	0.424	0.049	0.046	0.049	0.047	0.032
Model4	N3	0.886	0.719	0.665	0.681	0.665	0.048	0.061	0.054	0.066	0.042
Model1	N4	0.772	0.576	0.553	0.551	0.410	0.045	0.046	0.055	0.058	0.049
Model2	N4	0.719	0.549	0.508	0.497	0.380	0.062	0.047	0.064	0.061	0.053
Model3	N4	0.653	0.509	0.487	0.457	0.203	0.048	0.041	0.057	0.052	0.029
Model4	N4	0.738	0.549	0.498	0.513	0.420	0.051	0.050	0.049	0.050	0.042
Model1	N5	0.747	0.588	0.572	0.535	0.512	0.036	0.044	0.044	0.039	0.039
Model2	N5	0.711	0.535	0.515	0.488	0.478	0.064	0.066	0.052	0.046	0.028
Model3	N5	0.626	0.450	0.398	0.399	0.318	0.051	0.049	0.055	0.058	0.040
Model4	N5	0.713	0.525	0.504	0.494	0.497	0.048	0.049	0.056	0.052	0.048

FS, factor score meta-analysis; Mega, BFIM SEM mega-analysis; SS, mean score meta-analysis; T1, type I error rate.

As seen in **Figure 3**, the BFIM mega-analysis resulted in the most statistical power to detect the variant effect across all conditions. The advantage in relative power for the BFIM mega-analysis over the sum score ranged from about 4% (model 1, N4 condition) to about 19% (model 3, N1 condition). The BFIM mega-analysis also displayed larger power rates than the meta-analysis using harmonized factor scores. This is true even in the fourth data-generating model in which the BFIM is slightly misspecified. However, in this fourth model condition, the advantage of the BFIM mega-analysis over the sum score is generally at its smallest, compared to the other conditions.

The meta-analysis of factor scores also resulted in more statistical power than the meta-analysis of sum scores in 13 of the 20 different conditions. The power rates across these two methods were essentially equivalent in three conditions, and the sum score approach was more powerful in four conditions. The factor score meta-analysis resulted in more power for the first three data-generating models. However, with smaller sample sizes or unbalanced sizes across cohorts, the advantage of the factor score meta-analysis over the sum score is fairly small. In the fourth data-generating model, where the BFIM is slightly misspecified, the meta-analysis of factor scores is generally less powerful than sum score meta-analysis.

The multiple imputation approach used in these simulations resulted in the least power across nearly all conditions. This is especially true under data-generating model 3, when there are measurement differences across cohort, and in the fourth sample size condition, when there is significant imbalance in cohort size. For balanced sample sizes and no measurement differences across cohorts, the imputation approach performed similarly to the meta-analysis of sum scores. As seen in **Table 1**, all methods displayed acceptable type I error rates (between 2.5 and 7.5%).

### Bias and Coverage

**Figure 4:**
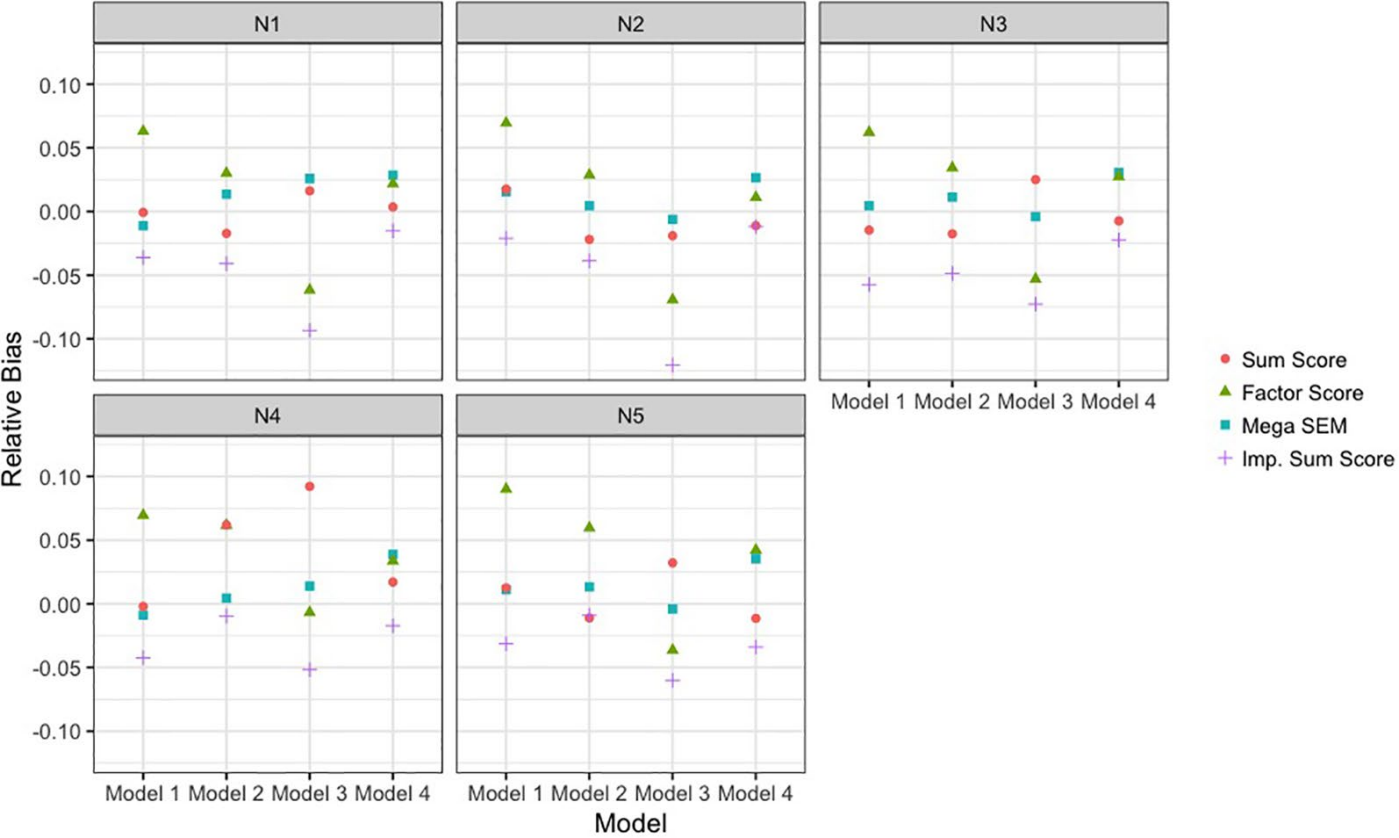
Empirical bias of single-nucleotide polymorphism effect of each analysis approach relative to true effect size. Results are presented across the different data-generating models and the different sample size conditions detailed in **Table 1**. The analysis approaches were sum score meta-analysis, factor score meta-analysis, mega-analysis with the bi-factor integration mode, and multiple imputation of the missing items. SEM, structural equation modeling; Imp., imputation.

**Figure 4** depicts the relative bias of the different methods across models and sample size conditions. Across all conditions, the BFIM mega-analysis estimates were within acceptable levels (±0.05) of relative bias. The bias in sum score meta-analysis fell within the acceptable range in 18 of the 20 conditions, and only presented problematic bias with unbalanced sample sizes. The bias resulting from factor score meta-analysis was between 5 and 10% in 8 of the 20 conditions, and was within acceptable levels in 12 of the 20 conditions. The imputation approach resulted in negative bias that was greater than 5% when the data-generating model was model 3. The sum score meta-analysis and the BFIM mega-analysis had excellent coverage rates, and the factor score meta-analysis had good coverage rates in 18 of the 20 conditions, as seen in **Table 3**.

**Table 3:** Relative bias, coverage rates, type I error rates, and standard errors computed with different analysis methods under the four data-generating models and five sample size conditions.

Model	N	Mega bias	FS bias	SS bias	Impute bias	Mega coverage	FS coverage	SS coverage	Impute coverage
Model1	N1	−0.011	0.063	−0.001	−0.036	0.944	0.939	0.962	0.972
Model2	N1	0.014	0.030	−0.017	−0.041	0.958	0.940	0.944	0.950
Model3	N1	0.026	−0.062	0.016	−0.093	0.959	0.903	0.948	0.966
Model4	N1	0.029	0.022	0.004	−0.015	0.949	0.941	0.956	0.955
Model1	N2	0.016	0.070	0.018	−0.021	0.941	0.926	0.937	0.947
Model2	N2	0.005	0.029	−0.022	−0.039	0.941	0.934	0.934	0.949
Model3	N2	−0.006	−0.069	-0.019	−0.121	0.941	0.910	0.944	0.963
Model4	N2	0.027	0.011	−0.011	−0.012	0.948	0.933	0.943	0.942
Model1	N3	0.005	0.062	−0.015	−0.057	0.952	0.929	0.949	0.962
Model2	N3	0.011	0.034	−0.017	−0.049	0.944	0.944	0.953	0.952
Model3	N3	−0.004	−0.053	0.025	−0.073	0.941	0.883	0.951	0.968
Model4	N3	0.031	0.027	−0.007	−0.022	0.958	0.944	0.950	0.966
Model1	N4	−0.009	0.070	−0.002	−0.042	0.954	0.934	0.956	0.966
Model2	N4	0.004	0.062	0.062	−0.010	0.957	0.938	0.949	0.960
Model3	N4	0.014	−0.007	0.092	−0.052	0.946	0.917	0.945	0.965
Model4	N4	0.039	0.034	0.017	−0.017	0.945	0.921	0.945	0.947
Model1	N5	0.011	0.090	0.012	−0.031	0.954	0.917	0.953	0.952
Model2	N5	0.013	0.060	−0.011	−0.009	0.955	0.929	0.950	0.958
Model3	N5	−0.004	−0.036	0.032	−0.060	0.938	0.917	0.936	0.961
Model4	N5	0.035	0.042	−0.011	−0.034	0.945	0.938	0.934	0.956

FS, factor score meta-analysis; Mega, BFIM SEM mega-analysis; SS, mean score meta-analysis; Impute, multiple imputation.

### Differences Across Simulation Conditions

The BFIM mega-analysis was most powerful and unbiased across all conditions. The pattern of results was also the same across the conditions, except with unbiased sample sizes across cohorts (N4). In this condition, the relative power of the BFIM mega-analysis increased as the models became more complicated, and the relative bias departed from zero more in this sample size condition.

The meta-analysis of factor scores was least effective under data-generating model 4. Under this model, the factor score meta-analysis was slightly less powerful than the sum score-meta analysis. In the other three models, the factor-score meta-analysis was generally more powerful, but the degree of advantage was largest with larger sample sizes.

### Advantage of the Reference Panel

The necessity of the reference panel was apparent in the simulations due to the low rates of convergence for both the BFIM mega-analysis and the BFIM factor analysis models. Without any overlapping items, the covariance matrix of the combined data had completely missing cells across the cohorts. When two items were treated as the same item, reflecting the real-world scenario of recoding items based on similar face validity, the BFIM models often did not converge. When estimates were obtained, the power of the BFIM mega-analysis was similar to the sum score meta-analysis, and the estimates were downwardly biased. The meta-analysis of factor scores resulted in very low rates of power, due in part to non-convergence across repetitions and to unstable estimates with large standard errors. **Table 4** provides details across the four data-generating models with the N1 condition when no reference panel was included.

**Table 4:** Results for sample size condition 1 when no reference panel data were included.

Model	N	Mega rel. power	FS rel. power	Mega bias	FS bias
mdl1	N1	0.690	0.465	−0.378	0.012
mdl2	N1	0.620	0.449	−0.426	0.158
mdl3	N1	0.531	0.410	−0.497	0.212
mdl4	N1	0.662	0.483	−0.228	0.189

### Results Summary

In conclusion, the BFIM mega-analysis approach employing the bi-factor integration model provided meaningful power increases, very low bias, and appropriate coverage. The factor score meta-analysis also resulted in power gains compared to sum score meta-analysis when the BFIM is correctly specified, although there was a small amount of bias in the estimates. Additionally, the use of the reference panel was crucial for the BFIM models. The measurement models were completely unstable when there was no item overlap, and harmonization carried out on two non-identical items caused issues for model estimation. The integration approach will be problematic when there is sparse item overlap, as would happen in consortia using different instruments across studies. The reference panel overcame this limitation and resulted in more power gain that the same amount of additional subjects added through one of the partners. Multiple imputation of the items using default settings followed by mega-analysis of sum scores resulted in the lowest power rates in all conditions.

## Application

The BFIM approach was demonstrated on data from two cohorts participating in the ACTION Consortium (http://www.action-euproject.eu/; Boomsma, 2015; Bartels et al., 2018). The main objective of ACTION is to improve understanding of the sources of individual differences in aggression among children to better inform treatment strategies. The ACTION consortium is unique because several participating cohorts used distinct questionnaires to measure aggression in children. The ACTION Consortium also collected reference panel data: parents (fathers and mothers) of young twins from the Netherlands Twin Register (van Beijsterveldt et al. (2013)) were contacted when their children were around 9 years old to complete supplemental questionnaires that were also administered among other partnering studies. The reference panel data facilitate harmonization using the BFIM approach. We demonstrate the data management and analysis plan for saving harmonized factor scores. These scores can then but used as the phenotype in any genetic analysis.

### Participants

For this application, data were analyzed from mother report for twins around age 9. In all cases, subjects were retained for analysis if they had less than 30% missing values on the aggression items administered within their cohort. Details of samples and measures used are below.

#### Netherlands Twin Register

For this study, 22,772 mother reports for the Netherlands Twin Register (NTR) were used from collections when children were approximately 9 (mean = 9.94, SD = 0.51). The sample was 50.4% female. For details on data collection in the NTR see e.g., van Beijsterveldt et al. (2013).

#### Child and Adolescent Twin Study in Sweden

Parents from the Swedish Twin Register were interviewed *via* telephone on the 9th birthday of their children. Mother report data were available for 18,278 children at age 9. The sample was approximately 49.4% female at age 9. For details on Child and Adolescent Twin Study in Sweden (CATSS) see Anckarsäter et al. (2011).

#### The Phenotypic Reference Panel

The reference panel is a supplemental collection of participants from the NTR with additional questionnaires collected. Throughout 2017, the complete survey items were collected. Questionnaires were mailed to families with children around age 9 (mean = 9.42, SD = 0.78). The current study utilized mother report data on 2,205 children. The reference panel is 51.5% female.

### Measures

#### Child Behavior Checklist

The Child Behavior Checklist (CBCL) 6–18 (Achenbach and Rescorla, 2001) was used by the NTR and the reference panel. The CBCL 6–18 consists of 120 items which are rated on a three-point scale ranging from “not true = 0,” “somewhat or sometimes true = 1,” to “very true or often true = 2.” In the CBCL 6–18 aggressive symptom subscale, we identified 8 items that pertain directly to an overt/physical subtype of aggression for this analysis (see Lubke et al., 2018). These items are listed in **Table 5**.

**Table 5:** Overt/physical aggression items in Aggression in Children: Unraveling gene-environment interplay to inform Treatment and InterventiON strategies.

Item code	Item
ATAC63	Has there ever been a time when he/she would be angry to the extent that he/she cannot be reached?
ATAC65	Does he/she often tease others by deliberately doing things that are perceived as provocative?
ATAC70	Has he/she ever been deliberately been physical cruel to anybody?
ATAC71	Does he/she often get into fights?
CBCL016	Cruelty, bullying, or meanness to others
CBCL020	Destroys his/her own things
CBCL021	Destroys things belonging to his/her family or others
CBCL023	Disobedient at school
CBCL037	Gets in many fights
CBCL057	Physically attacks people
CBCL094	Teases a lot
CBCL095	Temper tantrums or hot temper

#### Autism-Tics, Attention-Deficit Hyperactivity Disorder, and Other Comorbidities Inventory

The Autism-Tics, Attention-Deficit Hyperactivity Disorder, and Other Comorbidities Inventory (ATAC) (Larson et al., 2010) was administered in CATSS and the reference panel. The ATAC is a comprehensive screening interview for autism spectrum disorders, attention deficit/hyperactivity disorder, tic disorders, developmental coordination disorder, learning disorders, and other childhood mental disorders. The ATAC included four items related to overt/physical subtype of aggression, and responses were scored on a three-point scale (response options “yes,” “yes, to some extent,” and “no”). These items are listed in Table 5.

### Analysis Plan

**Figure 5:**
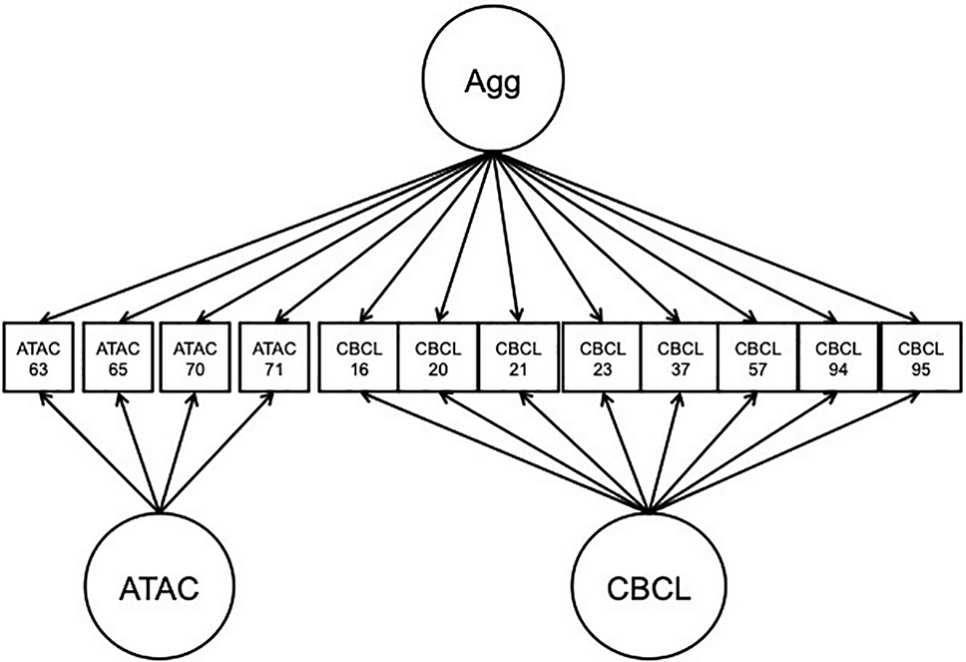
Path diagram of bi-factor integration model application to Aggression in Children: Unraveling gene-environment interplay to inform Treatment and InterventiON strategies data. Agg, overt aggression factor; CBCL, child behavior check-list; ATAC, autism-tics, attention-deficit hyperactivity disorder, and other comorbidities scale.

The NTR, CATSS, and reference panel data were concatenated into the same dataset for analysis. A BFIM was constructed in which all items were modeled by a general factor, representing the target trait of overt/physical type aggression. Specific factors were used to model ATAC-specific and CBCL-specific item subsets. The factors were specified to be standard normal, with all factor loadings freely estimated, and the factors were all uncorrelated with each other. The model is presented in **Figure 5**. Because subjects were nearly always twin pairings, sandwich-type robust standard errors were used for twins clustered within the same family. Analyses were carried out in Mplus 7.11 (Muthén and Muthén, 1998-2017).

## Results

The BFIM model overall displayed excellent fit to the data *χ* ^2 ^(42) = 418.98, *p *< 0.001, *RMSEA *= 0.014 [0.013, 0.016], *CFI *= 0.992, *TLI *= 0.988).While the sum of a limited number of categorical items provide a small number of possible observed scores, the factor scores provided more nuance based on the relationship among all items. The factor scores and the sum scores were correlated at 0.91. Factor scores were computed as maximum a posteriori estimates of the factor scores (the only option for categorical variables in Mplus). The harmonized scores could be returned to individual cohorts for genetic analyses prior to meta-analysis. If genetic data is shared among cohorts, the integration model could be used as part of a larger mega-analysis.

## Discussion

The current paper presented IDA as a phenotype scoring framework for combining data across multiple independent studies. A bi-factor model for data integration was proposed that was designed specifically to adjust for measurement differences across multiple cohorts such as the use of different questionnaires. A series of simulation studies compared the BFIM to the standard approach of using sum scores of available items in each cohort and demonstrated the benefits of IDA in terms of increased power to detect a SNP effect. The BFIM was applied to two partnering cohorts in the ACTION Consortium using the collection of a reference panel dataset with responses to the questionnaires from both cohorts.

The IDA approach presented here has implications for joint gene association analyses carried out in genetic consortia. Several reviews have emphasized the need to improve phenotype measurement and consistent phenotype definition across the individual studies participating in GWAS meta-analyses (Evangelou and Ioannidis, 2013; Robinson et al, 2014). Psychometric measurement models have been promoted in other areas of behavior genetics research as well, such as twin and family studies (van den Berg et al., 2007; van der Sluis et al., 2010; Schwabe and van den Berg, 2014; Luningham et al., 2017). Researchers have suggested item response models for use in multi-cohort studies of personality traits (van den Berg et al., 2014). The BFIM proposed in this paper is designed specifically for harmonization in association tests within a consortium of disparate studies.

Importantly, this study revealed that a factor score meta-analysis provided a gain in power over separate studies using sum score scores that were not directly comparable, provided the bi-factor model was adequately specified. This reflects the likely scenario where phenotypic data can be shared and jointly modeled in a consortium, but a full genome-wide search with SEM is not tenable. While the power gains found in this study were small in an absolute sense, the only difference in obtaining empirical power was in the method used. For GWAS analyses, where power is at a premium, a 4% gain in power simply through modeling the phenotype more precisely is a meaningful advantage. However, as advances such as GW-SEM (Verhulst et al., 2017) and genomic SEM (Grotzinger et al., 2019) make GWAS with multivariate outcomes and SEMs more feasible, our results suggest that the increase in power simply through IDA phenotypic modeling could be much greater.

A crucial aspect of the proposed IDA approach is the collection and use of a phenotypic reference panel. The concept of a phenotype reference panel to facilitate phenotype modeling was essential under our simulation scenarios. An important finding in the current paper is that the reference panel need only be a proportionately small increase in overall sample size to stabilize the integration model. It is more important to obtain a representative sample with complete phenotype information than to obtain new data as large as many of the participating partners. Collecting a small set of individuals that can bridge the gap in measurement items used across studies provide more benefit than increasing an individual cohort by the same amount of subjects.

The advantages of using a latent factor model to define a phenotype rather than a sum or mean score are reflected in the results of this paper. These advantages stem from using the full set of all available items and from accounting for different sources of heterogeneity in the observed score. The BFIM allows for modeling shared information in item subsets that does not pertain to the phenotype of interest, but to other sources of shared variance specific to certain studies. In our simulation, the residual covariance unrelated to the target trait was represented by different questionnaires. Additional sources of covariance among items can be incorporated into integrated measurement models, such as specific factors for different raters (e.g., mother and father) and residual covariance among similarly worded items. By modeling these as separate sources of commonality, the general factor becomes more precise. Covariate effects can also be included, such that item parameters differ as a function of age or gender (Curran et al., 2014).

More generally, the BFIM is not limited to GWAS, but can be applied in any joint analysis effort across multiple studies. IDA was proposed in psychological literature as a way to promote cumulative science, increase replicability of results, obtain broader psychometric assessments of constructs, and increase power (Curran and Hussong, 2009; Hussong et al., 2013). The bi-factor integration model presented in this paper is straightforward and has potential wide-ranging uses for detecting meaningful covariate effects on an integrated outcome. The bi-factor integration approach represents a potentially more powerful alternative to meta-analysis when phenotypic heterogeneity across studies needs to be taken into account.

The current study utilizes simulated and applied items that were already identified as pertaining to a unidimensional trait, in this case, overt aggression. An unexplored potential advantage of the IDA harmonization framework is the possibility to fit more complex models to larger item sets. Previous research has demonstrated that removing unreliable items in a questionnaire of a phenotype increases the power in a GWAS of that phenotype (Laurin et al., 2015). Further, conducting an association test of a sum score of items that actually originate from multiple subtypes of a trait can reduce GWAS power substantially compared to appropriately modeling the separate, but correlated, traits (van der Sluis et al., 2010). Applications of BFIM are contingent upon investigating the psychometric properties of the available questionnaires, and sum scores including irrelevant or unrelated items would likely lead to increasingly less power than an IDA approach. Future investigations can consider more complicated integration models.

In practice, the BFIM will need to be adapted to the particular data available. For example, one could utilize the bi-factor integration model and the multi-rater integration model of Bauer et al. (2013) to combine mother and father data across different studies in one analysis. Additionally, one could incorporate a limited number of covariate effects on item parameters as in the MNLFA model (Curran and Hussong, 2009; Curran et al., 2014). Consider a data integration scenario in which there were slight measurement differences across males and females. Rather than fitting separate models for each gender, one could use the bi-factor integration model and adjust for gender differences on individual items. Though real-world applications require careful application of complex measurement models, our study indicates that better phenotypic modeling with a reference panel can increase power at less cost than simply increasing sample size.

### Limitations

Our simulation design represents a fairly simplified scenario compared to the complexity of research designs in applied data. In real data applications, on the other hand, it is difficult to calculate exact power gains in a specific study because the true data-generating process in the measurement model is usually unknown. Furthermore, results from simulation studies are specific to the chosen conditions and do not necessarily generalize to all possible scenarios. The pattern of power gains found in this study is expected to hold whenever phenotype precision is improved. The multiple imputation procedure included for comparison in our simulation was also limited to using the default imputation model and limited settings. In practice, multiple imputation can employ a wide range of prediction models, and the procedure can be optimized by increasing iterations and/or the number of datasets. Our results should not be seen as an indictment on imputation itself but on the shortcomings of using only default settings. However, the BFIM is better suited to explicitly adjust for differences in item sets as they pertain to the true underlying phenotype score, compared to a composite score of imputed items.

Data integration approaches also face challenges and potential pitfalls. Data integration requires extensive data sharing efforts among collaborators. While data sharing is often quite streamlined in genetic consortia, such agreements are not the norm for joint research ventures. The social sciences in general may benefit by working more collaboratively across existing studies. Data integration requires extremely careful planning, and joint model-fitting efforts can be very complex. Furthermore, model fitting with a combined dataset that has a high degree of missingness requires computationally intensive estimation algorithms. On the other hand, computational resources are increasingly affordable, and models can be fitted with the help of distributed cluster computing and cloud storage. Finally, IDA is only effective if models are properly specified. Substantive experts and data analysts must work together closely to ensure that integrated phenotype models are theoretically sound.

Data integration is not a cure-all procedure to improve SNP detection, but it is a reasonable and additional step that can be taken in genetic collaborations to improve power in GWAS of complex phenotypes. With all of the effort in genetic research projects to collect genetic data, impute gene SNP information, control for genetic relatedness, and collaborate internationally, the additional effort in phenotype modeling is certainly a small price to pay for meaningful gains in power.

## Data Availability Statement

All datasets and scripts for the simulation study are included in the article/Supplementary Material. The NTR and CATSS datasets are not publicly available to protect sensitive phenotype information for participating children. The NTR and CATSS datasets are available by submitting a data request.

## Ethics Statement

Data were previously collected under approval of the participating cohorts’ original governing boards. All data used in the current analyses were collected under protocols that have been approved by the appropriate ethics committees, and studies were performed in accordance with the ethical standards laid down in the 1964 Declaration of Helsinki and its later amendments.

## Author Contributions

JL, DM, and GL devised the bi-factor approach for integration. JL designed and conducted simulation studies. DM and GL advised simulation design. JL and GL drafted the first manuscript. DB, MB and CB supervised NTR data collections and set up the reference panel. AH facilitated the merging of aggression data across multiple ACTION partners. PL, HL, and SL supervised CATSS data collection and its partnership in ACTION. All authors edited the manuscript.

## Funding

This work was supported by FP7-602768 “ACTION: Aggression in Children: Unraveling gene-environment interplay to inform Treatment and InterventiON strategies” from the European Commission/European Union Seventh Framework Program. GL was in addition supported by DA-018673 awarded by the National Institutes of Health: The funders had no role in study design, data collection and analysis, decision to publish, or preparation of the manuscript.

## Conflict of Interest

The authors declare that the research was conducted in the absence of any commercial or financial relationships that could be construed as a potential conflict of interest.

## Footnotes

1 We note that multiple imputation can utilize a large number of different prediction models, and the number of iterations and imputed datasets can be tuned and optimized across different settings. As this paper focuses on the explicit psychometric modeling and not imputation, we used the default model and a single set of imputation settings.

## Appendix I: Data-Generating Model Parameters

Four data-generating models were used in the simulations—three using a bi-factor model, and a fourth using a higher-order latent variable model that resulted in the bi-factor model having slight misspecification to the generated data. The vector of latent variables was generated from the following model for an individual *j*

(9) $$\eta_{j}=\;\beta x_{j}+\zeta_{j}$$

where η is a *k*×1 vector of *k* latent factors for person *j,* **x** **_j_** is a *Q* × 1 vector of *Q* predictors (the genetic variant and any covariates) for person *j,* β is a *k* × *Q* vector of effect sizes of each covariate on each latent factor, and z is a vector of residuals for the *k* factors. In these simulations, we generated a general factor that was predicted by a single nucleotide polymorphism (SNP) and a covariate term, along with two independent specific factors that did not have exogenous predictors:

(10) $$\begin{array}{l} \left(\begin{array}{c} \eta_{jg}\\ \eta_{j1}\\ \eta_{j2} \end{array}\right)=\left(\begin{array}{cc} 0.045 & 0.894\\ 0 & 0\\ 0 & 0 \end{array}\right)\left(SNP_{j}\;x_{j}\right)\;+\left(\begin{array}{c} \zeta_{jg}\\ \zeta_{j1}\\ \zeta_{j2} \end{array}\right),\\ \zeta_{j}\;\sim \;MVN\left(\left(\begin{array}{c} 0\\ 0\\ 0 \end{array}\right),\left(\begin{array}{ccc} .799 & 0 & 0\\ 0 & 1 & 0\\ 0 & 0 & 1 \end{array}\right)\right) \end{array}$$

In this equation, the residual variance of the general factor is 0.799 because it is calculated as 1 minus the sum of the variance explained by the predictors.

The item-level data is then generated from the factors and the factor loading matrices:

(11) $$y_{j}=\Lambda \eta_{j}+\varepsilon_{j}$$

where *y* *_j_* is a *p*×1 vector of items for person *j,* Λ is a *p*×*k* matrix of factor loadings for each *p* items on the *k* factors, and ɛ*_j_* is a *p*×1 vector of item residuals. To manipulate the conditions across the first three data-generating conditions, the loadings matrices and residual variance matrix was manipulated:

(12) $$\begin{array}{c} \Lambda_{1}=\left[\begin{array}{ccc} 0.6 & 0.49 & 0\\ 0.4 & 0.66 & 0\\ 0.5 & 0.59 & 0\\ 0.5 & 0.59 & 0\\ 0.6 & 0 & 0.49\\ 0.5 & 0 & 0.59\\ 0.5 & 0 & 0.59\\ 0.4 & 0 & 0.66 \end{array}\right];\Lambda_{2=}\left[\begin{array}{ccc} 0.5 & 0.45 & 0\\ 0.3 & 0.60 & 0\\ 0.4 & 0.54 & 0\\ 0.4 & 0.54 & 0\\ 0.6 & 0 & 0.49\\ 0.5 & 0 & 0.59\\ 0.5 & 0 & 0.59\\ 0.4 & 0 & 0.66 \end{array}\right];\\ \Lambda_{3}=\left[\begin{array}{ccc} 0.5 & 0.45 & 0\\ 0.3 & 0.60 & 0\\ 0.4 & 0.54 & 0\\ 0.4 & 0.54 & 0\\ 0.6 & 0 & 0.49\\ 0.5 & 0 & 0.59\\ 0.5 & 0 & 0.59\\ 0.4 & 0 & 0.66 \end{array}\right]; \end{array}$$

The residual variance is calculated as:

(13) $$Var\left(\varepsilon_{j}\right)\;\sim \;MVN\left(0,I_{p}-diag\left(\Lambda^{\prime }\Lambda \right)\right)$$

Meaning that the variance is a diagonal matrix. In addition, a covariance is inserted in the variance matrix such that *σ*_38_ = 0.6.

For the fourth data-generating model, the general factor is directly predicted by the SNP and covariate as before, and then the two cohort-specific factors are directly caused by the general factor itself, such that it accounts for 60% of the variance in the two factors:

(14) $$\begin{array}{l} \left(\begin{array}{c} \eta_{j1}\\ \eta_{j2} \end{array}\right)=\;\left(\begin{array}{c} 0.77\\ 0.65 \end{array}\right)\left(\begin{array}{c} \eta_{jg} \end{array}\right)+\left(\begin{array}{c} \zeta_{j1}\\ \zeta_{j2} \end{array}\right),\\ \;\zeta_{j}\;\sim \;MVN\left(\left(\begin{array}{c} 0\\ 0 \end{array}\right),\;I-diag\left(\left(\begin{array}{c} 0.77\\ 0.65 \end{array}\right)\left(\begin{array}{cc} 0.77 & 0.65 \end{array}\right)\right)\right) \end{array}$$

The item loading matrix is then *p*×2 instead of *p*×3 and the items also have a direct effect of the general factor. For graphical representations, see **Figure 2**. The codes used to conduct the simulation are attached as a supplementary downloadable folder.
